# Female sex as independent risk factor for chronic pain following elective incisional hernia repair: registry-based, propensity score-matched comparison

**DOI:** 10.1007/s10029-019-02089-2

**Published:** 2019-11-27

**Authors:** F. Köckerling, H. Hoffmann, D. Adolf, D.Weyhe, W. Reinpold, A. Koch, P. Kirchhoff

**Affiliations:** 1 Department of Surgery and Center for Minimally Invasive Surgery, Academic Teaching Hospital of Charité Medical School, Vivantes Hospital, Neue Bergstrasse 6, 13585 Berlin, Germany; 2 Center for Hernia Surgery and Proctology, St. Johanns-Vorstadt 44, 4056 Basel, Switzerland; 3 StatConsult GmbH, Halberstädter Strasse 40 a, 39112 Magdeburg, Germany; 4 Pius Hospital Oldenburg, Medical Campus of University of Oldenburg, University Hospital of Visceral Surgery, Georgstraße 12, 26121 Oldenburg, Germany; 5 Department of Surgery, Wilhelmsburger Hospital Groß Sand, Academic Teaching Hospital of University Hamburg, Groß Sand 3, 21107 Hamburg, Germany; 6 Hernia Center Cottbus, Gerhard-Hauptmann-Strasse 15, 03044 Cottbus, Germany

**Keywords:** Incisional hernia, Female sex, Chronic pain, Hernia registry, Postoperative complications, Recurrence

## Abstract

**Introduction:**

To date, little attention has been paid by surgical scientific studies to sex as a potential influence factor on the outcome. Therefore, there is a sex bias in the surgical literature. With an incidence of more than 20% after 3 years, incisional hernias are a common complication following abdominal surgical procedures. The proportion of women affected is around 50%. There are very few references in the literature to the influence of sex on the outcome of elective incisional hernia repair.

**Materials and methods:**

In all, 22,895 patients with elective incisional hernia repair from the Herniamed Registry were included in the study. The patients had undergone elective incisional hernia repair in a laparoscopic IPOM, open sublay, open IPOM, open onlay or suture technique. 1-year follow-up was available for all patients. Propensity score matching was performed for the 11,480 female (50.1%) and 11,415 male (49.9%) patients, creating 8138 pairs (82.0%) within fixed surgical procedures.

**Results:**

For pain on exertion (11.7% vs 18.3%; *p* < 0.001), pain at rest (7.53% vs 11.1%; *p* < 0.001), and pain requiring treatment (5.4% vs 9.1%; *p* < 0.001) highly significant disadvantages were identified for the female sex when comparing the different results within the matched pairs. That was also confirmed on comparing sex within the individual surgical procedures. No sex-specific differences were identified for the postoperative complications, complication-related reoperations or recurrences. Less favorable intraoperative complication results in the female sex were observed only for the onlay technique.

**Conclusions:**

Female sex is an independent risk factor for chronic pain after elective incisional hernia repair.

## Introduction

To date, little attention has been paid by surgical scientific studies to sex as a potential influence factor on the outcome [1, 2]. Therefore, there is sex bias in the surgical literature [1]. The Surgery Journal Editors Group has published a statement specifying that in the future sex should be considered as a biologic variable in all studies [3, 4].

With an incidence of up to 22.4% after three years, incisional hernias are a common complication following abdominal surgical procedures [5–7].

In registry studies there is essentially no difference between the proportion of men and women in the total collective of incisional hernias, with each accounting for 50% [8–11].

There are very few references in the literature to the influence of sex on the outcome of elective incisional hernia repair. Data from healthcare cost and utilization project—nationwide inpatient sample—on 59,993 elective ventral- and incisional hernia-repairs showed higher mortality for the male sex [12]. A prospective study of 887 ventral- and incisional hernia-patients from the International Hernia Mesh Registry revealed an increased risk of chronic pain for the female gender [13].

The following study of data from the Herniamed Hernia Registry [14, 15] aimed to show differences in the outcome of male vs female patients following elective incisional hernia repair.

## Materials and methods

The Herniamed quality assurance study is a multicenter, internet-based hernia registry [14, 15] into which 712 participating hospitals and surgeons (Herniamed Study Group) in Germany, Austria, and Switzerland (status: February 1, 2019) have entered data prospectively on their patients who had undergone routine hernia surgery [16]. All patients signed an informed consent agreeing to participate [16]. As part of the information provided to patients regarding participation in the herniamed hernia registry and signing the informed consent declaration all patients are informed that the treating hospital or medical practice would like to be informed about any problem occurring after the operation and that the patient has the opportunity to attend clinical examination [17].

All postoperative complications occurring up to 30 days after surgery were recorded [16]. At 1-year follow-up, postoperative complications were once again reviewed when the general practitioner and patient completed a questionnaire [16]. At 1-year follow-up, the general practitioner and the patient were also asked about any recurrences, pain at rest, pain on exertion, and chronic pain requiring treatment [16]. The relevance of patient reported outcomes after incisional hernia repair was demonstrated [18].

The present retrospective analysis of prospective data compares the perioperative and one-year follow-up data of female and male patients who underwent elective incisional hernia repair between September 1, 2009 and January 1, 2018. The surgical procedures included in the analysis were the laparoscopic-intraperitoneal onlay mesh (IPOM), open sublay, IPOM, and onlay techniques as well as open-suture repair. Inclusion criteria were a valid minimum age of 16 years, elective-incisional hernia repair, use of approved meshes on the market, and complete registry database entry in mandatory fields, including complete 1-year follow-up.

Physiomesh was excluded from this analysis because of its voluntary market withdrawal [19]. For enhanced comparability, recurrences were also excluded.

All statistical analyses were performed using the software SAS 9.4 (SAS Institute Inc., Cary, NC) and intentionally calculated to a full significance level of 5%, i.e., they were not corrected with respect to multiple testing, and each *p* ≤ 0.05 represents a significant result.

Propensity score matching was performed using greedy algorithm and a caliper of 0.1 standard deviations. The variables used for matching were as follows:

American Society of Anesthesiologists (ASA) status, age, body mass index (BMI, kg/m^2^), preoperative pain, incisional hernia defect size (W1 ≤ 4 cm, W2≧4–10 cm, W3 > 10 cm), and defect localization (medial, lateral, combined) according to the European Hernia Society classification [20], mesh size (cm^2^), use of drain (yes vs no), risk factors (chronic obstructive pulmonary disease, diabetes mellitus, aortic aneurysm, immune suppression, cortisone medication, smoking, coagulopathy, anticoagulant, or antiplatelet medication), and operative procedure. The latter had to be identical within each of the pairs.

The balance of the matched sample was assessed using standardized differences (also given for the pre-matched sample), which should not exceed 10% (< 0.1) after creating matched pairs. For pairwise comparison of matching parameters between female and male patients (to present the differences between the original pre-matched samples), *χ*^2^ test and *t* tests (Satterthwaite) were performed for categorical and continuous variables, respectively. Mesh size had been log-transformed beforehand due to the strong deviation from the normal distribution.

Matched samples were then analyzed for perioperative and 1-year follow-up outcomes (intra- and postoperative complications, complication-related reoperations, pain at rest and on exertion, pain requiring treatment, and recurrences) via McNemar’s test. The results obtained are presented as the non-diagonal elements of the 2 × 2 frequency table, the corresponding p values and the odds ratio (OR) estimates for matched samples with 95% confidence interval.

## Results

**Fig. 1:**
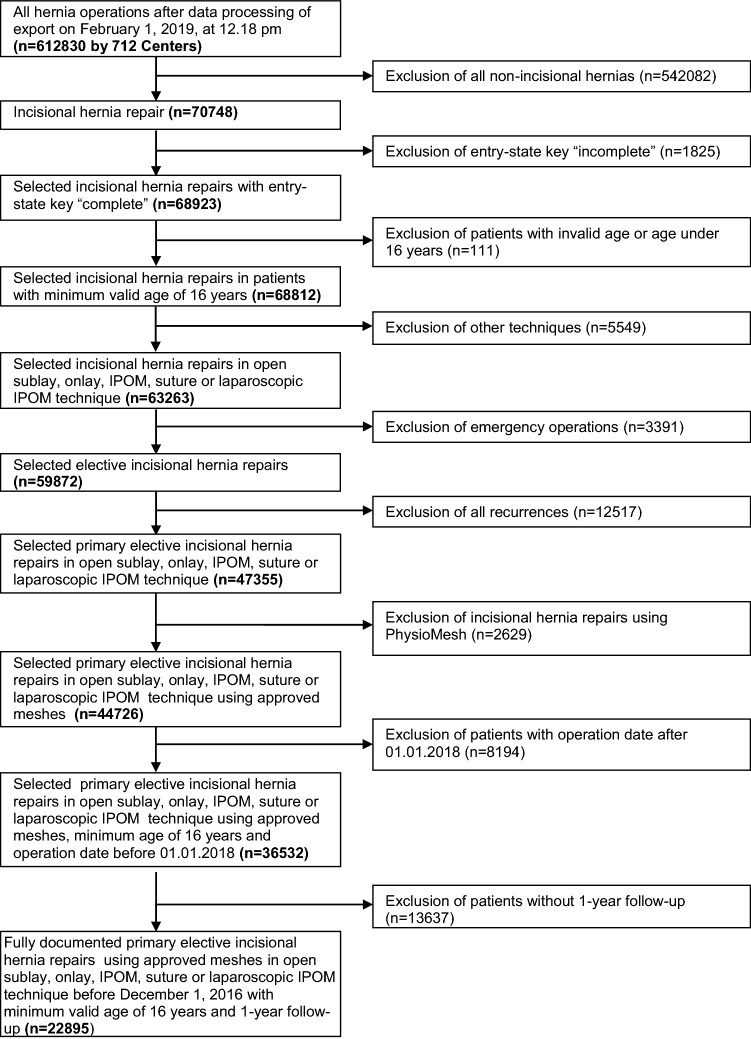
Flowchart of patient inclusion

In all, 22,895 patients were included in this retrospective analysis (Fig. 1). Of the 22,895 patients, 11,480 were women (50.1%) and 11,415 men (49.9%). Propensity score matching was performed for these 22,895 patients to obtain homogeneous comparison groups [21].

### Unadjusted analysis

Comparison of the influence factors before matching showed significant differences between men and women (Table 1). For women suture techniques were used more often, they had fewer ASA classifications III/IV, more frequent preoperative pain, fewer risk factors, higher BMI (male vs female: 27.7 ± 4.8 vs 29.6 ± 6.6; *p* < 0.001) and a smaller mesh had been used for them (male vs female: 241.1 cm^2^ [238.7; 243.6] vs 222.8 cm^2^ [220.5; 225.2]; *p* < 0.001). No significant difference in age (male vs female: 62.8 ± 12.6 years vs 63.2 ± 14.2 years; *p* = 0.069) or drain placement (male vs female: 55.9% vs 53.97%; *p* = 0.091) was identified between men and women.

**Table 1:** Patient and procedure related influencing factors on the outcome

	Sex				*p*
	Male		Female		
	*n*	%	*n*	%	
Procedure					
Laparoscopic-POM	3139	27.50	3222	28.07	< 0.001
Open suture	1105	9.68	1557	13.56	
Open IPOM	1692	14.82	1504	13.10	
Open Onlay	622	5.45	676	5.89	
Open Sublay	4857	42.55	4521	39.38	
ASA-score					
I	1267	11.10	1336	11.64	< 0.001
II	6333	55.48	6637	57.81	
III/IV	3815	33.42	3507	30.55	
EHS-classification (width)					
W1 (< 4 cm)	4132	36.20	4483	39.05	< 0.001
W2 (> = 4 - 10 cm)	5245	45.95	5274	45.94	
W3 (> 10 cm)	2038	17.85	1723	15.01	
EHS-classification (defect localisation)					
Lateral	1855	16.25	2112	18.40	< .001
Medial	8589	75.24	8461	73.70	
Combined	971	8.51	907	7.90	
Preoperative pain					
Yes	5827	51.05	7261	63.25	< .001
No	4615	40.43	3244	28.26	
Unknown	973	8.52	975	8.49	
Drainage					
Yes	6288	55.09	6196	53.97	0.091
No	5127	44.91	5284	46.03	
*Risk factors*					
Total					
Yes	5078	44.49	4304	37.49	< .001
No	6337	55.51	7176	62.51	
COPD					
Yes	1147	10.05	1199	10.44	0.323
No	10,268	89.95	10,281	89.56	
Diabetes					
Yes	1427	12.50	1565	13.63	0.011
No	9988	87.50	9915	86.37	
Aortic aneurism					
Yes	306	2.68	64	0.56	< .001
No	11,109	97.32	11,416	99.44	
Immunosuppression					
Yes	216	1.89	190	1.66	0.174
No	11,199	98.11	11,290	98.34	
Corticoids					
Yes	168	1.47	221	1.93	0.008
No	11,247	98.53	11,259	98.07	
Smoking					
Yes	1628	14.26	1119	9.75	< .001
No	9787	85.74	10,361	90.25	
Coagulopathy					
Yes	289	2.53	186	1.62	< .001
No	11,126	97.47	11,294	98.38	
Antiplatelet medication					
Yes	1688	14.79	1041	9.07	< .001
No	9727	85.21	10,439	90.93	
Anticoagulation therapy					
Yes	410	3.59	302	2.63	< .001
No	11,005	96.41	11,178	97.37	

### Matched pairs analysis

Propensity score matching was performed using greedy algorithm and a permitted caliper width of 0.1 standard deviations for the 11,415 male and the 11,980 female patients. Matching was performed for *n* = 8138 (82.0%) patients.

**Fig. 2:**
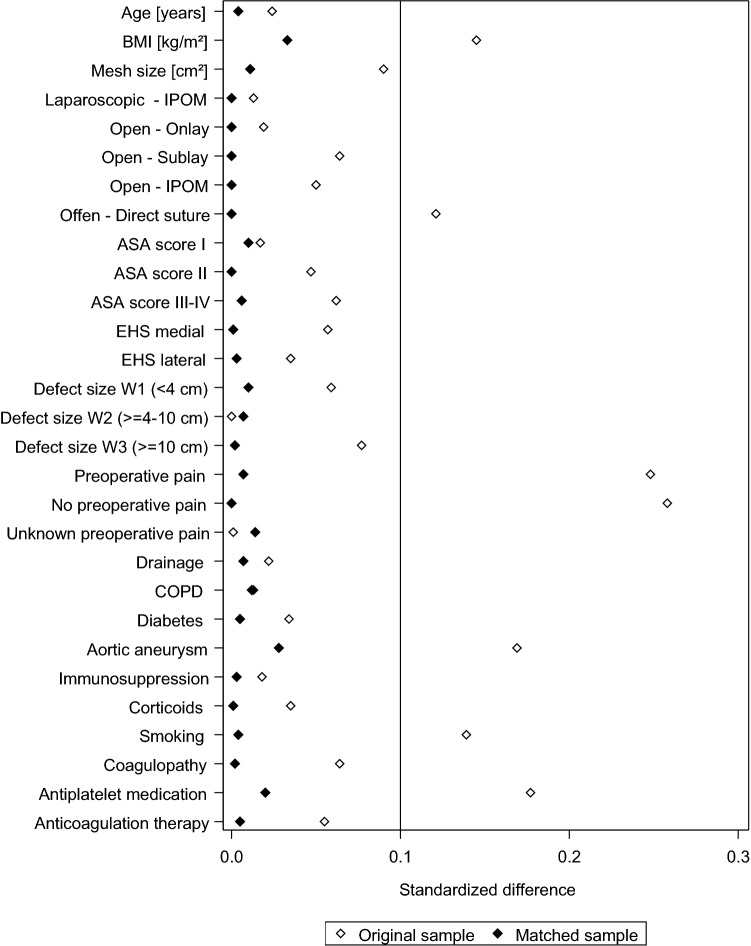
Standard differences between the matching variables both before (original sample) and after matching (matched sample)

Figure 2 shows the standard differences between the matching variables both before (original sample) and after (matched sample) matching.

That difference was well below 10% for all matching variables, attesting to a good balance between the variables included in the model.

**Fig 3:**
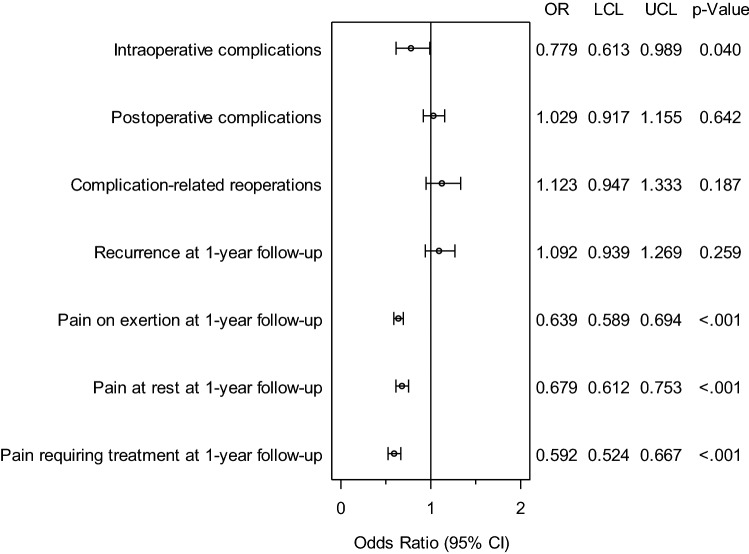
Results of matched pairs analysis of incisional hernia repair in female vs male patients

Figure 3 and Table 2 give a summary of the sex-specific results for the various outcome parameters. A search for systematic differences in the results for male vs female patients was undertaken. A systematic difference was identified between the sex groups for the intraoperative complications and the pain rates at 1-year follow-up. For the intraoperative complications, in addition to concordant complication cases for four pairs (0.05%), a significant deviation was identified in favor of the male patients (1.6% vs 2.0%; *p* = 0.040). Likewise, for pain at rest, affecting 65 pairs (0.8%) in the case of both men and women, highly significant disadvantages were noted for women (7.53% vs 11.1%; *p* < 0.001). The same was true for pain on exertion (11.7% vs 18.3%; *p* < 0.001 for an additional 284 pairs (3.5%) with concordant pain) and pain requiring treatment (5.4% vs 9.1%; *p* < 0.001), with the latter pain occurring additionally in 39 pairs (0.5%) in both cases. No significant differences were seen between men and women in the postoperative complications, complication-related reoperations, general postoperative complications or the recurrence rates.

**Table 2:** Results of matched pair analysis of incisional hernia repair in female vs male patients

	Disadvantage		*p* value	OR for matched samples		
	Male	Female		OR	Lower limit	Upper limit
Intraoperative complications	1.56	2.00	0.040	0.779	0.613	0.989
General complications	3.19	3.61	0.161	0.884	0.746	1.049
Postoperative complications	7.40	7.19	0.642	1.029	0.917	1.155
Complication-related reoperation	3.59	3.19	0.187	1.123	0.947	1.333
Recurrence on 1-year-follow-up	4.53	4.15	0.259	1.092	0.939	1.269
Pain on exertion on 1-year-follow-up	11.67	18.26	< .001	0.639	0.589	0.694
Pain in rest on 1-year-follow-up	7.53	11.10	< .001	0.679	0.612	0.753
Chronic pain requiring treatment on1-year-follow-up	5.36	9.06	< .001	0.592	0.524	0.667

### Matched pairs analysis of the different surgical procedures

To identify more accurately the role of sex as an independent influence factor on the outcome of individual surgical procedures too, matched pairs analyses were performed additionally for these subgroups. To that effect, the outcomes for men versus women were compared for 3837 pairs with open-sublay repair, 2475 pairs with laparoscopic-IPOM repair, 535 pairs with open-onlay repair, and 1267 pairs with open-IPOM method. These analyses were omitted for open-suture repair since that applied to only 24 pairs. Below only the discordant matched-pairs outcomes are reported.

For the open-sublay repair highly significant differences (*p* < 0.001) to the disadvantage of women were identified for pain on exertion (men vs women: 12.1% vs 18.3%), pain at rest (men vs women: 7.8% vs 11.2%), and pain requiring treatment (men vs women 5.3% vs 9.2%. For the intraoperative complications no significant difference (men vs women: 1.3% vs 1.5%; *p* = 0.444) was found (Table 3).

**Table 3:** Results of matched pair analysis of incisional hernia repair with sublay technique in female vs male patients

	Disadvantages		*p* value	OR for matched samples		
	Male	Female		OR	Lower limit	Upper limit
Intraoperative complication	0.75	2.99	0.012	0.250	0.061	0.775
General complication	1.68	4.86	0.006	0.346	0.143	0.762
Postoperative complication	9.35	9.35	1.000	1.000	0.662	1.511
Complication-related reoperation	5.05	2.99	0.126	1.687	0.877	3.353
Recurrence on 1-year-follow-up	4.67	6.54	0.245	0.714	0.410	1.228
Pain on exertion on 1-year-follow-up	11.59	19.81	< 0.001	0.585	0.420	0.808
Pain in rest on 1-year-follow-up	7.10	13.08	0.003	0.543	0.356	0.817
Pain requirement treatment on 1-year-follow-up	6.36	9.35	0.101	0.680	0.426	1.072

For the laparoscopic-IPOM repair comparable outcomes were noted. Likewise, for pain on exertion (men vs women: 11.4% vs 18.3%), pain at rest (men vs women: 6.7% vs 10.8%), and chronic pain requiring treatment (men vs women: 4.5% vs 8.9%) highly significant differences (*p* < 0.001) were identified to the disadvantage of women. For laparoscopic-IPOM repair no difference was seen in the intraoperative complications (men vs women: 2.0% vs 2.4%; *p* = 0.391) (Table 4).

**Table 4:** Results of matched pair analysis of incisional hernia repair with laparoscopic IPOM technique in female vs male patients

	Disadvantages		*p* value	OR for matched samples		
	Male	Female		OR	Lower limit	Upper Limit
Intraoperative complications	2.02	2.42	0.391	0.833	0.561	1.233
General complications	2.55	2.30	0.648	1.105	0.760	1.610
Postoperative complications	3.19	3.72	0.359	0.859	0.628	1.173
Complication-related reoperation	1.58	1.49	0.909	1.054	0.655	1.700
Recurrence on 1-year-follow-up	4.00	4.08	0.944	0.980	0.735	1.306
Pain on exertion on 1-year-follow-up	11.43	18.26	< 0.001	0.626	0.538	0.728
Pain in rest on 1-year-follow-up	6.71	10.79	< 0.001	0.622	0.509	0.757
Pain requiring treatment on 1-year-follow-up	4.53	8.85	< 0.001	0.511	0.404	0.645

For the open-IPOM repair only for pain on exertion was a highly significant influence observed to the disadvantage of women (men vs women: 10.9% vs 17.5%; *p* < 0.001). For pain at rest (men vs women: 8.5% vs 10.7%; *p* = 0.095), and chronic pain requiring treatment (men vs women: 7.0% vs 9.1%; *p* = 0.068) only a negative trend was identified for women. Likewise, for the open-IPOM repair no difference was found in the intraoperative complications between men and women (men vs women: 1.7% vs 2.1%; *p* = 0.568).

For the open-onlay repair female gender was found to have a highly significant negative influence on the outcome for pain on exertion (men vs women: 11.6% vs 19.8%; *p* < 0.001), a significantly negative influence for pain at rest (men vs women: 7.1% vs 13.1%; *p* = 0.003) and a negative trend towards chronic pain requiring treatment (men vs women: 6.4% vs 9.4%; *p* = 0.101). For the open-onlay repair a disadvantage was also identified for women in the intraoperative complications (men vs women: 0.8% vs 3.0%; *p* = 0.012).

### Influence of BMI

In a subgroup analyses of the matched pairs population comparing the subgroup of patients with and without reported pain in the 1-year follow-up no significant difference in the mean BMI was found in male (29.27 vs 29.15 kg/m^2^; *p* = 0.395) and female (28.95 kg/m^2^ vs 29.01 kg/m^2^; *p* = 0.710) patients.

### Sub-group of patients without follow-up

**Fig. 4:**
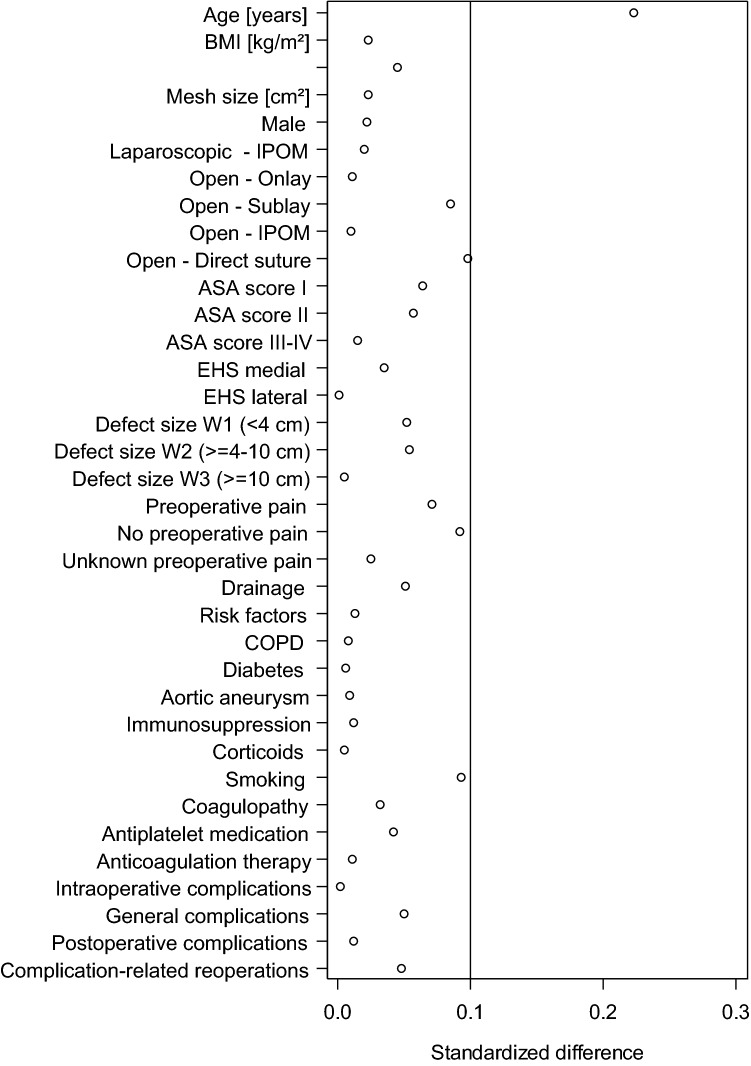
Standardized differences of the influencing factors and the perioperative outcomes between patient collectives with and without follow-up

To exclude selection bias of patients, an additional analysis of the sub-group of patients without 1-year follow-up was performed. With the exception of age with a mean difference of 2.4 years all other influencing factors and perioperative outcomes had a standardized difference below 0.1 compared to the patients with 1-year follow-up (Fig. 4). Therefore, both collectives are comparable which excludes selection bias. The higher age in the sub-group without follow-up demonstrates more difficulties in getting patient reported outcome information from the older patients.

## Discussion

Since, to date, only very few studies in the surgical literature have considered sex as a biological variable, the Journal Editors Group has called upon surgical scientists to publish more sex-specific studies [1–4]. Whereas for inguinal hernia sex differences were only recently recompiled [16, 22], there is a paucity of publications on the influence of sex on the outcome after ventral- and incisional hernia-repair [12, 13]. The large number of cases in registries are eminently suited to comparative studies of men vs women [23] since no patients are excluded and, at best, all other potential influence factors on the outcome are also taken into account.

For the 8138 pairs of opposite sex but with similar distribution of the other influence variables, in the present analysis highly significant differences were found in the rates of pain on exertion, pain at rest, and chronic pain requiring treatment at 1-year follow-up to the disadvantage of the female gender. That finding was confirmed, too, in a subgroup analysis in which propensity score-matched pairs were also compared for the individual surgical procedures, and in which larger subsamples may yield higher power as a matter of course. For the most common surgical techniques, in particular, i.e., the open-sublay repair and laparoscopic-IPOM repair, female sex was found to have a highly significantly negative effect on chronic pain on exertion, pain at rest and pain requiring treatment. In a further subgroup analysis no influence of a higher BMI on the postoperative chronic pain rates could be found. This thus demonstrates that sex is an independent, unfavorable influence factor for chronic pain rates after incisional hernia repair.

In a series of 887 ventral hernia repairs Cox et al. [13], too, found female sex to be associated with a higher risk of chronic pain.

“Chronic or long-term pain following open-incisional hernia repair is poorly documented. Traditionally, studies of incisional hernia repair have focused only on short-term complications (infection, hematoma), major morbidity, hospital stay, and recurrence” [24].

For inguinal hernia a systematic review revealed that female gender had a significant influence on the rate of chronic inguinal pain after inguinal hernia repair [25].

In another review of chronic postoperative pain, in addition to preoperative pain, psychological factors (e.g., anxiety, depression, catastrophizing), younger age, surgical factors (e.g., open approach, length of operation > 3 h), and intensity of pain in immediate postoperative period, female sex was also implicated in causation [26].

The analysis results of primary elective incisional hernia repair based on data from the Herniamed Hernia Registry thus confirm the findings in the literature indicating that female sex is an independent risk factor for chronic pain. That relationship can be statistically demonstrated, in particular, for the most commonly performed surgical procedures, i.e., laparoscopic-IPOM- and open-sublay repair. But for open IPOM and onlay repair, too, higher pain rates are identified for women with otherwise influence variables similar to men.

“Men and women differ in their responses to pain, with increased pain sensitivity and risk of clinical pain commonly being observed among women” [27]. “After decades of assuming that pain works the same way in all sexes, scientists are finding that different biological pathways can produce pain” [28].

Female patients should therefore be informed about the higher risk of getting chronic postoperative pain in the preoperative counseling.

Matters are different as regards the intraoperative complications. Here, overall analysis reveals a slightly significant disadvantage for women. But that disadvantage can no longer be identified when comparing the individual surgical procedure subgroups for laparoscopic IPOM, open sublay, and open IPOM. Only for the onlay technique is a significant disadvantage observed for women. Hence, that disadvantage of female gender can be demonstrated only for the onlay technique. For the much more common surgical techniques, i.e., open sublay and IPOM as well as laparoscopic IPOM, no negative influence is seen.

Registry studies have a number of limitations. A relevant proportion of patients had to be excluded from analyses because no follow-up data were available. But the sub-group analysis does not show any suspicion of selection bias. Incorrect or missing data limit a registry [16]. Comparison with other registry or literature data is only possible to an extent due to the lack of publications. However, the findings reported here concord with the limited data available in the literature.

In conclusion, female sex is an independent risk factor for chronic pain after elective incisional hernia repair. That holds true for the surgical procedures laparoscopic IPOM, open sublay, open IPOM, and open onlay. Because of the small number of cases no conclusive statement can be made about open suture repair. No demonstrable disadvantage could be identified for female gender with regard to the perioperative complications, complication-related reoperations or recurrence rate.
